# The RAPid COmmunity COGnitive screening Programme (RAPCOG): Developing the Portuguese version of the Quick Mild Cognitive Impairment (Q*mci*-P) screen as part of the EIP on AHA Twinning Scheme

**Published:** 2019-01-06

**Authors:** P Machado dos Santos, R O’Caoimh, A Svendrovski, C Casanovas, F Orfila Pernas, M Illario, W Molloy, C Paul

**Affiliations:** 1 Center for Health Technology and Services Research/ICBAS, Institute of Biomedical Sciences Abel Salazar – University of Porto, Porto, Portugal; 2 Health Research Board, Clinical Research Facility Galway, National University of Ireland, Galway, Ireland; 3 Centre for Gerontology and Rehabilitation, University College Cork, St Finbarr’s Hospital, Douglas Road, Cork City, Ireland; 4 UZIK Consulting Inc., 86 Gerrard St E, Unit 12D, Toronto, ON, M5B 2J1 Canada; 5 Institut Universitari d’Investigació en Atenció Primaria Jordi Gol (IDIAP JGol), Barcelona, Spain; 6 Division on Health Innovation, Campania Region Health Directorate; DISMET/R&D Unit, Federico II University & Hospital, Naples, Italy

**Keywords:** screening, cognitive impairment, community, program, Quick Mild Cognitive Impairment Screen

## Abstract

As populations age and the prevalence of cognitive impairment increases, healthcare professionals and researchers require short, validated cognitive screening instruments (CSIs). As part the EIP-on-AHA Twinning Support Scheme (2016), four reference sites developed the RAPid COmmunity COGnitive screening Programme (RAPCOG) twinning project to validate translated versions of the Quick Mild Cognitive Impairment (Q*mci*) screen that could be adapted quickly for use with future eHealth screening and assessment programmes. Here we present the cultural adaption and translation of the Q*mci*-Portuguese (Q*mci*-P) screen as part of RAPCOG and explore its subsequent validation against two commonly-used CSIs (MMSE-P and MoCA-P) with 93 participants aged ≥65, attending ten day care centres or resident in two long-term care institutions; median age 74 (+/−15), 66% female. The Q*mci*-P’s internal consistency was high (Cronbach’s Alpha 0.82), compared with the MoCA (0.79) and SMMSE (0.54). Q*mci*-P screen scores moderately correlated with the SMMSE (r=0.61, 95% CI:0.45–0.72, p<0.001) and MoCA (r=0.63, 95% CI:0.36–0.80, p<0.001). The Q*mci*-P screen demonstrates high internal consistency and concurrent validity against more established CSIs and given its brevity (3–5mins), may be preferable for use in community settings. This project shows the potential of the EIP-on-AHA Twinning initiative to promote the scaling-up of innovative good practices.

## I. INTRODUCTION

Dementia is a growing and important public health concern [1, 2] associated with an increased risk of adverse healthcare outcomes [2], elevated expenditure [3], and a greater number of years lived and lost with disability [4]. Multi-dimensional intervention strategies introduced before the onset of functional impairment may slow cognitive decline [5]. Although there is insufficient evidence to support routine cognitive screening among asymptomatic older adults [6, 7], identifying those with subjective decline and mild cognitive impairment (MCI) may be important [8, 9].

Currently, there is no consensus on which screening instrument should be used to detect cognitive impairment [7], particularly MCI [10], though healthcare professionals express a preference for brief and easy-to-use cognitive screening instruments (CSI) [11, 12]. The Quick Mild Cognitive Impairment (Q*mci*) screen (www.qmci.ie), a short (3–5 minutes) CSI designed to differentiate normal cognition from MCI and early dementia [13], is sensitive across the spectrum of cognitive impairment [14, 15, 16] and is validated in multiple settings, countries and languages [17–24]. It may be an ideal CSI to rapidly screen and triage older adults for further assessment. Despite this, it is not translated or validated in many European Union (EU) countries.

The European Innovation Partnership on Active and Healthy Ageing (EIP-on-AHA), launched in 2010, aims to achieve a triple win of improved health and quality of life for older adults, sustainable health systems and an enhanced and competitive healthcare marketplace [25]. Dedicated action groups under its umbrella have created good practice initiatives called commitments with the A3 Action Group focused on the prevention of ageing related frailty, diseases and functional decline [26–28]. Since its inception, the EIP-on-AHA has fostered the development of reference sites and synergies between these to develop a network of interconnected sites across Europe dedicated to achieving its aims [29–31]. In 2016, it launched its’ Twinning Support Pilot Scheme to promote the scaling-up of good practices between reference sites, bringing together 43 twinning organisations from 13 countries through a series of projects [31].

This study presents the results of the translation, refinement and initial validation of the Q*mci* screen in Portugal as part of the RAPid COmmunity COGnitive screening Programme (RAPCOG) twinning project developed by four EIP-on-AHA reference sites: Ireland’s Collaboration on Ageing (COLLAGE) [32] as originator and the Metropolitan Area of Porto (Porto4Ageing; Portugal) [33] as, Campania area reference site (Italy) (34) and the Catalonia reference site (Barcelona, Catalonia, Spain) [35] as adopters. Its overarching goal was to adapt and develop an existing CSI for use with any future cognitive screening and assessment programmes, particularly one that could be computerised to support eHealth screening.

## II. METHODOLOGY

### Overview (Milestones) of the RAPCOG Twinning Project

Trainers (the developers of the Q*mci* screen) from the originator site in Ireland travelled to two adopter sites (Porto in October 2016 and Barcelona in February 2017) to provide training to local staff and partners from the other two adopter sites. This involved a defined review of existing structures and systems in the originator country, education sessions and workshops with staff. Face-to-face meetings with clinic and community-based staff were also conducted. Milestones were set for trialling the translated version and initiating validation in the adopter sites – a central step in showing that the instrument and information technology (IT) application are acceptable and accurate for use in the adopter sites. Translation of the Q*mci* screen followed by back-translation happened in advance of the site visits (described in detail below; Milestone 1). These were discussed and deliberated upon during the face-to-face meeting, which served to identify local and cultural issues with adaption, adoption and implementation after which a plan (protocol) was accepted (Milestone 2). The originator site continued to support the validation process and provide logistical, statistical and expert clinical support for each site (Milestones 3–6). Sampling and trialling in the field then proceeded in each site (Milestones 3–6). Follow-up meetings were scheduled for the mid-point of the initiative (interim – progress meeting – Milestone 5) and at the end (Milestone 6). The final meeting was held in the originator site in Ireland in June 2017.

Here we present the outcomes from the Portuguese site. A similar, concurrent approach to translating and validating Spanish and Catalan versions was conducted in primary care in Barcelona and will be reported elsewhere once data collection is complete (327 participants included to date).

### Participants

RAPCOG took advantage of a planned study to examine the clinical effectiveness of brief screening instruments for use in community settings called the Instrumentos Breves para Idosos (IBIS) study [36]. IBIS was designed to compare the construct validity of Portuguese language versions of the Q*mci* screen (Q*mci*-P), the Mini-Mental State Examination (MMSE-P) [37] and the Montreal Cognitive Assessment (MoCA-P) [38, 39]. Scores were also compared with measures of activities of daily living (ADL), personality and mood. The validation study was conducted with older adults aged ≥65 years, attending ten day care centres (n=113) and residents (n=53) in two long-term care institutions in Porto, Portugal who were included using convenience sampling. Participants provided consent to take part and those who completed screening were then invited to complete questionnaires. Demographic data (age, gender and education) as well as clinical data on cognition, personality, depression and functional status were collected during each assessment. Participants provided informed consent and the study received ethics approval in advance. The IBIS study protocol included the following measures:

### Outcome measures

Cognition was screened and assessed using the Standardised MMSE-P (SMMSE-P) [37] and the MoCA-P [38, 39]. The recently translated Q*mci*-P (see Appendix) was also used to investigate criterion-related (concurrent) validity. The Q*mci*-P screen has six subtests: orientation (10 points), five word registration (5 points), a clock drawing test (15 points), one-minute delayed recall (20 points), verbal category (semantic) fluency and logical memory, a test of immediate verbal recall of a short story [16, 40] to a total score of 100 with an established cut-off of 62/100 for cognitive impairment (MCI or dementia) [41]. It is a short CSI with a median administration time of 4.24 minutes [40]. It has superior accuracy to the 6-item CIT [14] and the SMMSE [13, 18] and is non-inferior but with a shorter administration time compared to the MoCA [15, 21, 23]. It also has moderate to strong correlation with the Lawton and Brody ADL scale and global measures of cognition such as the Clinical Dementia Rating scale and Alzheimer’s Disease Assessment Scale-cognitive section [42].

In addition to tests of cognition, the Neo-FFI 20 personality inventory was scored [43]. This assesses the “Big Five” personality traits: Extraversion, Agreeableness, Conscientiousness, Neuroticism, and Openness to Experience. The Portuguese Version of the Geriatric Depression Scale (GDS-P) [44, 45] was used to screen for significant depressive symptoms and generate a score that classifies participants as “normal” (0–10),”mildly depressed” (11–20) or “severely depressed” (21–30). Participants who scored 21 or more on the 30-point GDS (signifying likely moderate-severe depression), were excluded. Health status was measured using the EQ-5D-3L [46], a standardised measure that provides a simple five-item descriptive profile and a single index value, the EQ visual analogue scale (EQ-VAS) from 100-0, where the endpoints are labelled “Best imaginable health state” and “Worst imaginable health state”, respectively.

### Translation of the Qmci-P

The Q*mci*-P was translated into Portuguese by neuropsychologists with a good understanding of English and by a bilingual English teacher. This version was then edited and culturally adapted by a bilingual Portuguese-English speaker, without knowledge of the concepts behind the screening tool, to produce a second iteration. This was then back-translated to English, using the inverse method [47] by another bilingual clinical neuropsychologist. The back-translated version was sent to authors for review and later discussed at a research panel meeting including the authors of this paper (see https://www.linkedin.com/pulse/rapcog-pedro-machado-dos-santos). Suggestions and edits were incorporated at the RAPCOG twinning meeting in Porto to create version 3.

Consensual validation was then performed using a Portuguese Delphi panel, fluent in English, who assessed and compared the different versions in terms of semantic, idiomatic and conceptual equivalent of the items’ contents. If there was no consensus, the majority of the five panel members ruled on any issue. However, there was consensus on all issues resulting in the definitive version of the Q*mci*-P screen used in this study. A pre-test was performed with a sample of five persons during clinical consultation who reported that there were no issues with the contents of the statements.

### Statistical analysis

Data were analysed using SPSS version 24.0. The Shapiro–Wilk test, performed to test for normality, found that the majority of data were non-parametric. Correlations were determined using Spearman’s rho with bootstrapping for non-parametric data. The Kruskal-Wallis H test compared three or more non-normally distributed variables. Cronbach’s Alpha was used to measure internal consistency of the CSIs.

## III. RESULTS

Overall, 166 people were approached of whom 148 agreed to participate and were screened with the Q*mci*-P. These had a median age of 77 years, interquartile range (IQR) +/−15 and 64% were female. Of these 148 participants, 103 completed the full assessment battery with the remainder withdrawing stating time constraints or fatigue as reasons. Participants completing the assessment battery were then scored on the GDS and those scoring ≥21, indicating possible active depression, were excluded (n=11) leaving a final sample of 93 for analysis. These had a median age of 74 years (IQR +/−15), significantly younger than all those initially consenting (p=0.03). Their demographics and other characteristics are presented in Table 1.

**Fig. 1:**
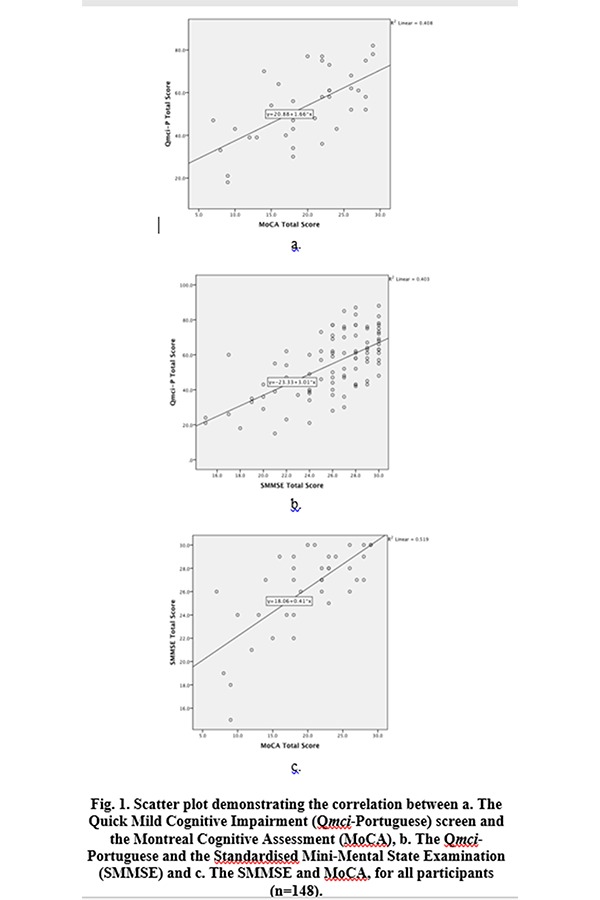
Scatter plot demonstrating the correlation between a. The Quick Mild Cognitive Impairment (Q*mci*-Portuguese) screen and the Montreal Cognitive Assessment (MoCA), b. The Q*mci*-Portuguese and the Standardised Mini-Mental State Examination (SMMSE) and c. The SMMSE and MoCA, for all participants (n=148).

The median Q*mci*-P screen score of those included was 57/100 (IQR +/−26) with a median MoCA of 21/30 (IQR +/−8) and median SMMSE of 27/30 (IQR +/−5). Q*mci*-P screen scores strongly, positively and significantly correlated with both the SMMSE (r=0.61, 95% confidence interval 0.45–0.72, p<0.001) and the MoCA (r=0.63, 95% confidence interval 0.36–0.80, p<0.001). The correlation between the SMMSE and MoCA was also strong (r=0.67, 95% confidence interval 0.43–0.84, p<0.001). Scatter plots are presented in Figure 1. Internal consistency of the Q*mci*-P screen measured using Cronbach’s Alpha was 0.82. This compared with 0.79 for the MoCA and only 0.54 for the SMMSE. The median GDS score of those included was 10 +/−10 points. There was a gradient effect associated with participant GDS scores with statistically significant differences between the median Q*mci*-P screen scores for those with GDS scores of 0–14 versus 15–20 and ≥21 (Q*mci*-P screen scores of 58, 47 and 35 respectively, χ^2^=11, p=0.004), see Table 1. This was similar for the SMMSE (χ^2^=7.6, p=0.02) but not the MoCA (χ^2^=3, p=0.23).

Using the established cut-off for the Q*mci* screen, <62/100 [41], the majority (n=58, 62%) of the sample screened positive for cognitive impairment (MCI or dementia). This compared to 76% with the MoCA using the widely used cut-off of <26 (38), which fell to 62% when a lower cut point designed to improve diagnostic accuracy of <23 was selected. Only 19 (20%) participants screened positive for cognitive impairment with the SMMSE at its established cut-off of <24 [37]. The proportion of participants screening positive for cognitive impairment with each of the cognitive screening instruments at different published cut-offs is presented in Table 2.

Immediately after completion of the final meeting held in the originator site, partners experiences of the Twinning project were discussed to finalise the report on the Twinning Activity for the European Commission. In summary, the three adopter sites collectively reported that cultural differences between the countries were a major challenge in translating the instruments in a way that the results would be consistent between sites. Round table discussion through the forum of the twinning support scheme was really valued by all. Face-to-face discussion facilitated these nuanced discussions akin to a mini Delphi consensus panel. In addition, challenges were reported with recruiting sufficiently trained staff to validate the instrument in each of the adopter countries resulting in the need to bring in additional staff from other sites. Further, it is expected that additional resources in terms of funding will be required to fully incorporate the translated versions into an IT application. Partners skillsets were predominantly clinical and other personnel with IT and business acumen are now required.

## IV. DISCUSSION

The main goals of this study were to report on the EIP-on-AHA RAPCOG Twining initiative (2016–17) involving four reference sites geographically dispersed across the EU (Ireland, Portugal, Spain – Catalonia - and Italy) (31), which aimed to adapt the Q*mci* screen as a brief CSI for use in any future community-based cognitive screening programmes, particularly those that could be adapted for use with existing and future eHealth IT infrastructure. Here we present the results of the translation and initial validation of the Q*mci*-P for use in Portuguese-language countries, exploring its concurrent validity against the most commonly used short CSIs in Portugal, the MMSE-D and the MoCA-P. The translation and adaptation of Q*mci* screen resulted in the development of a Portuguese version that is conceptually equivalent to the original. That is, the instrument is natural and acceptable and performs in the same way with an emphasis on cross-cultural and conceptual, rather than on linguistic/literal equivalence.

The strength of this study is the robust analysis used to identify the discriminatory characteristics of the Q*mci*-P screen in comparison to other CSIs, providing more accurate results than non-bootstrapped methods, especially when analysing smaller sample sizes. The 95 % confidence intervals obtained from the bootstrap and the asymptotic approach, were in all cases virtually equal. This indicates that the intervals are valid. The Q*mci*-P screen demonstrates high internal consistency with a Cronbach’s Alpha of 0.82, higher than that of the other instruments and in keeping with other studies of the Q*mci* screen (23, 42). This study also presents its concurrent validity against more established CSIs showing moderate, positive and significant correlation with the MoCA-P. However, given its brevity (3–5mins), (10, 15), it may be preferable for use in community settings.

This case exemplar shows the potential of the Commissions’ Twinning Support Scheme to facilitate the rapid up-scaling of a good practice initiative and an existing commitment under the EIP-on-AHA. Review of the project after the final meeting showed that all participants were satisfied with the process, though concerns were expressed, particularly in relation to how a lack of IT expertise among an academic and clinical research group to realise the potential of the instrument as an eHealth tool. The results, nevertheless show the potential of such a scheme to produce rapid results. Once fully validated and implemented in all the languages of the participating reference sites, it is hoped that the new solution will help streamline cognitive screening assessments in the community in each of the adopted sites. This is expected to save time, resources and money if evidence-based treatments for dementia emerge, strengthening the as yet limited evidence for community-based cognitive screening (6, 7). Irrespective, it is expected that it may lead to improved screening (case-finding) pathways with more patients receiving prompt and timely diagnosis. Since this project ended, new opportunities have arisen following discussions with other twinning sites linked to the adopters (e.g. Naples, Campania site in Italy is also twinned to a reference site in Croatia in a different twinning initiative. The Croatian site in Zagreb has now agreed to participate area by translating and validating the Q*mci* screen into Croatian (Q*mci*-Cro):, see https://ec.europa.eu/eip/ageing/commitments-tracker/a3/validation-croatian-version-quick-mild-cognitive-impairment-screen-qmci-cro-0_en. In addition, an EU-funded project called ProEmpower plans to use the Q*mci* screen in four pilot sites – Turkey, Portugal, Campania and Murcia.

The study has limitations. First, the diagnosis of MCI and dementia were not based on clinical criteria but on a battery of assessments, potentially misclassifying participants. However, the purpose was not to correlate the tools with clinical diagnoses but to examine the feasibility of using them in this population of older adults and examine their concurrent validity. Participants were recruited by convenience sampling. This could have created selection bias. Few community-dwellers were recruited (only those attending day care centres) potentially reducing external generalizability. Finally, the validation sample was small likely underpowering the study.

## V. CONCLUSION

As health professionals and researchers are faced with a growing older population but yet limited assessment time and clinical resources, there is a need to develop short CSIs for clinical and public health practice. The RAPCOG Twinning initiative shows the potential of the EIP-on-AHA reference sites to quickly up-scale good practice as highlighted by the development and validation of the Q*mci*-P screen as part of this pilot scheme. The Q*mci*-P had moderate, positive correlation with two short CSIs, commonly used in Portugal, but given its brevity (3–5 minutes), it may be preferable for use with older adults than the MMSE (7–8 minutes) and the MoCA (10–12 minutes) (15). Further research is now required to examine the psychometric properties of the instrument.

## 



## Figures and Tables

**Table 1:** Socio-demographic characteristics of the final sample included (n=93).

Variable	Level	Number	Proportion (% of total)	Total Median (Q3-Q1*±IQR)	Qmci Screen Median (Q3-Q1*±IQR)	MoCA Median (Q3-Q1*±IQR)	SMMSE Median (Q3-Q1*±IQR)
Age(Years)	All	93	100	74 (82-67*±15)	57 (69-43*±26)	21 (24-16*±18)	27 (29-24*±5)
	≤74	48	52	67 (71-66*±5)	61 (69-45*±24)	22 (26-18*±8)	28 (29-26*±3)
	75–84	31	33	79 (83-76*±7)	57 (71-46*±25)	18 (22-9*±13)	27 (28-24*±4)
	≥85	14	15	87 (88-86*±2)	43 (55-37*±18)	10 (11-9*±2)	24 (26-21*±5)
Gender	Male	32	34	-	61 (75-50*±25)	23 (27-20*±7)	28 (29-27*±2)
	Female	61	66	-	50 (63-39*±24)	18 (23-14*±9)	26 (28-24*±4)
Educational Level (years)	All	82	100	4 (6-4*±2)	-	-	-
	0–5	58	71	4 (4-3*±1)	58 (64-41*±23)	22 (25-18*±7)	27 (28-24*±4)
	6–11	17	21	8 (10-6*±4)	63 (75-55*±20)	21 (28-20*±8)	29 (30-27*±3)
	≥12	7	8	15 (15-13*±2)	67 (77-36*±41)	14 (16-11*±5)	28 (29-24*±5)
Geriatric Depression Scale (30 points)	All	93	100	9 (13-5*±8)	-	-	-
	0–14	76	82	7 (11-4*±7)	58 (71-43*±28)	20 (26-16*±10)	27 (29-24*±5)
	15–20	17	18	17 (18-15*±3)	47 (60-39*±21)	21 (22-18*±4)	26 (28-24*±4)
	≥21*	11	-	22 (24-22*±2)	35 (45-29*±)	10 (14-10*±4)	23 (26-21*±5)

* Excluded data (n=11)

Qmci screen = Quick Mild Cognitive Impairment screen

MoCA = Montreal Cognitive Assessment

SMMSE = Standardised Mini-Mental State Examination

**Table 2:** Comparison of the proportion of participants screening positive for cognitive impairment using established cut-offs for the Quick Mild Cognitive Impairment (Q*mci*-Portuguese) screen, Montreal Cognitive Assessment (MoCA) and Standardised Mini-Mental State Examination (SMMSE).

Variable	Median for total sample	Cut-off for Cognitive Impairment (MCI or dementia)	Proportion Normal (%)	Proportion Impaired (%)	Cut-off reference paper
Q*mci*-P screen	57 (69-43^*^26)	<62	35 (38)	58 (62)	(41) O’Caoimh et al., 2017
		<65 (MCI only)	27 (29)	66 (71)	(41) O’Caoimh et al., 2017 (15) O’Caoimh et al., 2016
MoCA	21 (24-16^*^±8)	<26	9 (24)	28 (76)	(38) Nasreddine et al., 2005
		<24	10 (27)	27 (73)	(48) Damian et al., 2011
		<23	14 (38)	23 (62)	(49) Luis et al., 2009 (50) Carson et al., 2017
		<22 (MCI only)	18 (49)	19 (51)	Freitas et al., 2013
MMSE or SMMSE	27 (29-24^*^±5)	<28	39 (42)	54 (58)	(41) O’Caoimh et al., 2017
		<24	74 (80)	19 (20)	(37) Morgado et al., 2009

IQR = Interquartile Range; MCI = Mild Cognitive Impairment; Q = Quartile
