# Supplementary figures and images for: The RAPid COmmunity COGnitive screening Programme (RAPCOG): Developing the Portuguese version of the Quick Mild Cognitive Impairment (Qmci-P) screen as part of the EIP on AHA Twinning Scheme
Source: Transl Med UniSa. 2019 Jan 6;19:82–9. (PMC6581493)

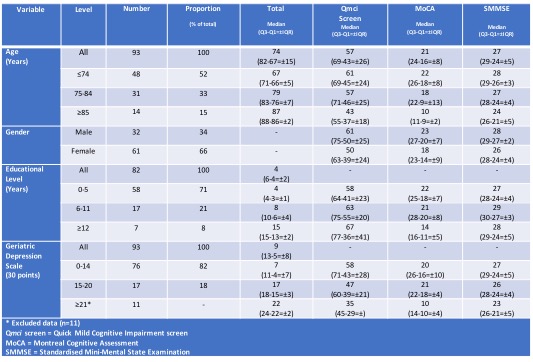


**Table 1. Socio-demographic characteristics of the final sample included (n=93).**

Supplement: Supplementary file 1 [file TM-19-082-s001.doc]
